# Programmable sequential mutagenesis by inducible Cpf1 crRNA array inversion

**DOI:** 10.1038/s41467-018-04158-z

**Published:** 2018-05-15

**Authors:** Ryan D. Chow, Hyunu Ray Kim, Sidi Chen

**Affiliations:** 1 Department of Genetics, Yale University School of Medicine, New Haven, CT 06511 USA; 2 Systems Biology Institute, Yale University School of Medicine, West Haven, CT 06516 USA; 3 Medical Scientist Training Program, Yale University School of Medicine, New Haven, CT 06511 USA; 4 Biological and Biomedical Sciences Program, Yale University School of Medicine, New Haven, CT 06511 USA; 5 Immunobiology Program, Yale University School of Medicine, New Haven, CT 06511 USA; 6 Comprehensive Cancer Center, Yale University School of Medicine, New Haven, CT 06511 USA; 7 Stem Cell Center, Yale University School of Medicine, New Haven, CT 06511 USA

## Abstract

Mutations and genetic alterations are often sequentially acquired in various biological and pathological processes, such as development, evolution, and cancer. Certain phenotypes only manifest with precise temporal sequences of genetic events. While multiple approaches have been developed to model the effects of mutations in tumorigenesis, few recapitulate the stepwise nature of cancer evolution. Here we describe a flexible sequential mutagenesis system, Cpf1-Flip, with inducible inversion of a single crRNA array (FlipArray), and demonstrate its application in stepwise mutagenesis in murine and human cells. As a proof-of-concept, we further utilize Cpf1-Flip in a pooled-library approach to model the acquisition of diverse resistance mutations to cancer immunotherapy. Cpf1-Flip offers a simple, versatile, and controlled approach for precise mutagenesis of multiple loci in a sequential manner.

## Abstract (web summary)

Few models recapitulate the stepwise nature of cancer evolution. Here the authors introduce Cpf1-Flip, a system to introduce mutations sequentially through inducible inversion of a crRNA array.

## Introduction

In a large variety of biological and pathological processes, genetic mutations or alterations are often acquired in a sequential manner^1–3^. In evolution and speciation, the genomes of organisms acquire mutations constantly and are subjected to natural selection^4^. In genetically complex disorders such as cancer, multi-step mutagenesis is often a major obstacle for effective treatments. Cancers evolve through an ongoing process of mutation–selection balance, where initial mutations are selected for, or against, in vivo, followed by subsequent acquisition of additional mutations as the tumor grows^5^. Since the initial set of oncogenic “driver” mutations is generally what starts and sustains tumor growth, targeted molecular therapies are often chosen to specifically attack such oncogenic dependencies^5^. However, the selection pressures of treatment will favor secondary mutations that confer drug resistance, leading to relapse^6–8^. Thus, the process of cancer evolution by sequential mutagenesis stymies these therapies via continuous diversification and adaptation to the tumor microenvironment, eventually exhausting available treatment options^5^. Even with the advent of cancer immunotherapy, where checkpoint blockade is increasingly being utilized in the clinic, the acquisition of secondary mutations that abolish T cell receptor (TCR)–antigen–major histocompatibility complex recognition can still lead to immune escape and ultimately negate the effect of immunotherapy^9,10^. Thus, the ability to perform sequential and precise mutagenesis is critical for studying biological processes with multi-stage genetic events such as development and evolution, as well as the pathogenesis of complex diseases such as cancer.

From a genetic engineering perspective, stepwise mutagenesis or perturbation is a powerful technique for precise genetic manipulation of cells and live organisms. Multiple methods have been employed to achieve this end. In the pre-recombinant DNA era, stepwise perturbation was often done by multiple rounds of random mutagenesis using chemical or physical carcinogens followed by artificial selection. The subsequent discovery and application of recombinase systems such as Cre-*loxP*, Flp-*FRT*, and φC31-*att* enabled inducible genetic events^11^. In these systems, the DNA recombinase (i.e., Cre) specifically recognizes its target DNA sequence motif (i.e., *loxP*) and catalyzes recombination between two such target sites. Depending on the configuration of the target sites, targeted recombinases can be utilized for DNA excision, translocation, and/or inversion. However, the floxed genomic loci underlying Cre-based systems must be pre-engineered on a gene-by-gene basis. This process of generating new floxed alleles for each unique application is time and labor intensive, further limiting the feasibility of multiplexed Cre recombination.

More recently, precisely targeted and customizable mutagenesis was simplified by the discovery of RNA-guided endonucleases (RGNs) Cas9^12–14^ and Cpf1^15,16^ from clustered regularly interspaced short palindromic repeats (CRISPR) systems. RGNs can induce double-strand DNA breaks, subsequently generating insertions and deletions at the target site. This process is precisely targeted based on the sequences of CRISPR RNAs (crRNAs), which complex with RGNs to enable and guide their nuclease functions. Unlike with Cre recombination, CRISPR crRNAs can be easily transferred to target cells through transfection or viral vectors, thus obviating the need to pre-engineer the host genome for each target gene.

In contrast to Cas9, the most widely utilized RGN to date, Cpf1 is a single component RGN that does not depend on trans-activating RNA and can autonomously process crRNA arrays^15–17^. These features have made Cpf1 particularly attractive for multiplexed mutagenesis. In addition to several studies in mammalian systems, Cpf1-mediated mutagenesis and transcriptional repression have now been successfully applied in plants^18–20^. Furthermore, chemical modifications on Cpf1 mRNA and crRNAs have been identified that can improve cutting efficiency^21^. It was recently demonstrated that Cpf1 can also process crRNAs from mRNAs expressed by a Pol II promoter^22^, further enabling flexible transcriptional control.

Sequential mutagenesis using Cas9 was demonstrated in ex vivo organoid cultures^23^. However, this approach required sequentially introducing each sgRNA in culture, one at a time, limiting its broader applicability. In particular, the sequential introduction of different sgRNAs would be impractical for library-scale screening or any in vivo experimental designs. To our knowledge, conditional sequential mutagenesis using RGNs has not yet been demonstrated. Here we describe a flexible sequential mutagenesis system through inducible inversion of a single crRNA array (Cpf1-Flip) and demonstrate its simplicity in stepwise multiplexed gene editing in mammalian cells for modeling sequential genetic events, such as in cancer. We further apply Cpf1-Flip to model the acquisition of resistance mutations to immunotherapy in a pooled mutagenesis setting, demonstrating the feasibility of Cpf1-Flip for conducting sequential genetic studies. This system can be utilized for multi-step mutagenesis of any genes in the genome for interrogating complex genetic events with temporal control.

## Results

### Construction of a Cpf1 sequential mutagenesis system

When *loxP* sites are arranged such that they point towards each other, Cre recombination leads to inversion of the intervening sequence. However, this process leads to the complete regeneration of the *loxP* sites, thereby allowing Cre to continually catalyze DNA inversion. As continuous Cre-mediated inversion would be counterproductive in many applications, mutant *loxP* sites have been characterized that enable unidirectional Cre inversion^24,25^. When the mutant *loxP* sites *lox66* and *lox71* are recombined, they generate a wild-type *loxP* site and a double-mutant *lox72*. Cre has a substantially lower affinity for *lox72*, thus leading to mostly irreversible inversion of the floxed DNA segment.

**Fig. 1:**
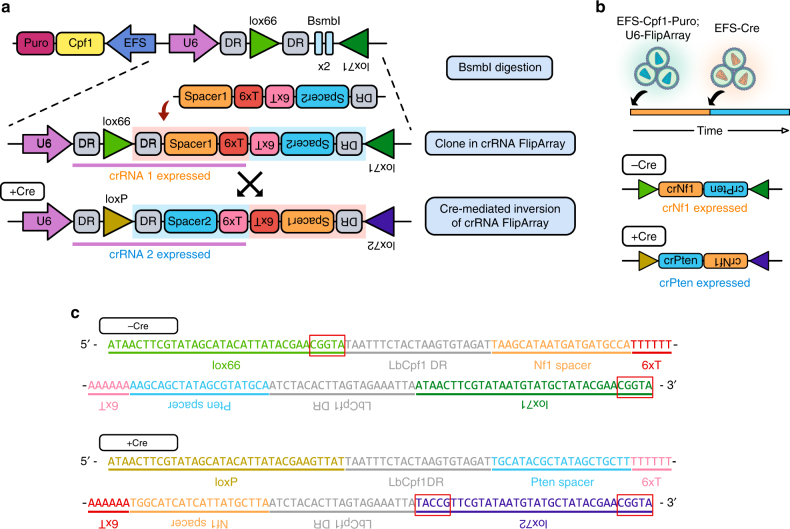
Cpf1–Flip–Cre-inducible sequential mutagenesis by a single crRNA FlipArray. **a** Schematic of vectors used in the study. The Cpf1-Flip construct contains an EFS promoter driving expression of Cpf1 and puromycin resistance, and a U6 expression cassette containing two inverted BsmbI restriction sites, flanked by a *lox66* sequence and an inverted *lox71* sequence. After BsmbI digestion, a crRNA FlipArray is cloned in. The FlipArray inverts upon Cre recombination, thereby switching the crRNA that is expressed. **b** Schematic of experimental design. Cells were first infected with lentivirus containing EFS-Cpf1-puro; U6-FlipArray. After 7 days, cells were then infected with lentivirus containing EFS-Cre to induce inversion of the FlipArray. Prior to Cre recombination, only crNf1 is expressed; following Cre recombination, crPten becomes expressed. **c** Sequences of the FlipArray construct before and after Cre recombination. Red boxes denote mutants from wild-type *loxP*. Prior to Cre, single mutant *lox66* and *lox71* sites are present. After Cre recombination, a wild-type *loxP* site and a double mutant *lox72* site are generated

We designed a U6 expression cassette containing two inverted BsmbI restriction sites, flanked by a *lox66* sequence and an inverted *lox71* sequence (Fig. 1a). In the same lentiviral vector, an EFS promoter drives the expression of *Lachnospiraceae bacterium* Cpf1 (LbCpf1, or Cpf1 for short)^15^ and a puromycin resistance gene (EFS-Cpf1-Puro). After BsmbI restriction digest, the vector linearizes and allows for insertion of a crRNA array. To enable stepwise mutagenesis, we designed crRNA arrays in which the first crRNA is encoded on the sense strand, while the second crRNA is inverted. We hereafter refer to this construct as a crRNA FlipArray. Six consecutive thymidines (6xT) are present in *cis* at the 3′ end of each crRNA, terminating U6 transcription. Each crRNA is preceded by the LbCpf1 direct repeat (DR) sequence, which guides Cpf1 to process the crRNA array^16^.

Cre-mediated recombination of the *lox66* and *lox71* mutant *loxP* sites^24,25^ leads to inversion of the FlipArray, generating a wild-type *loxP* and a double-mutant *loxP*, *lox72*. As the affinity of Cre recombinase for *lox72* is substantially lower than for wild-type *loxP*, inversion of the FlipArray is mostly irreversible^24,25^. After inversion, the two crRNAs trade places and the second crRNA becomes expressed. Thus, in the absence of Cre, Cpf1 generates indels at the target site of the first crRNA; after Cre recombination, Cpf1 is directed to the target site of the second crRNA. We hereafter term this approach Cpf1-Flip. In short, the Cpf1–Flip system leverages CRISPR-Cpf1 mutagenesis and melds it with the inversion capabilities of Cre/*lox66*/*lox71* to enable programmable two-step mutagenesis.

### Sequential mutagenesis in murine and human cells

To demonstrate sequential editing of cancer genes, we first applied Cpf1-Flip to generate *Nf1* and *Pten* mutations in a mammalian lung cancer cell line (KPD)^26^. We cloned in a FlipArray containing a spacer targeting Nf1 (crNf1) and an inverted spacer targeting Pten (crPten) (crNf1-crPten FlipArray, or NPF). We infected the cells with lentivirus containing EFS-Cpf1-Puro; U6-NPF (Fig. 1b). The pre-recombination construct was designed to only express crRNA targeting the first locus (*Nf1*) prior to the introduction of Cre. After 6 days of puromycin selection (1 week after the initial lentiviral transduction), we then infected the cells with lentivirus containing an EFS promoter driving the expression of Cre (EFS-Cre). Cre-expressing cells undergo inversion of the crRNA FlipArray, leading to sequential mutagenesis at the second locus (*Pten*) (Fig. 1c).

**Fig. 2:**
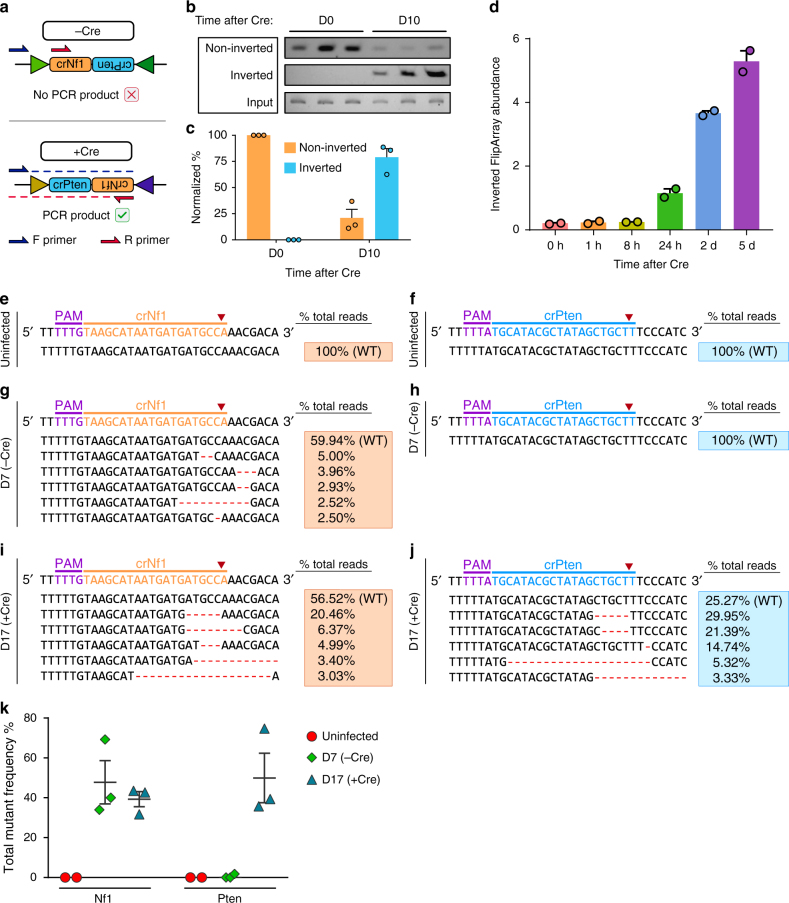
Inducible sequential mutagenesis in murine cells through Cpf1-Flip. **a** Schematic for PCR-based detection of Cre-mediated inversion of the crRNA FlipArray (targeting *Nf1* and *Pten*). **b** PCR-based detection of non-inverted and inverted FlipArrays at D0 (*n* = 3) and D10 (*n* = 3) following Cre, along with input control. **c** Quantification of gel intensities in **b**, normalized to input and expressed as a percentage of total FlipArray abundance. **d** Detection and quantification of Cre-mediated inversion of the crRNA FlipArray at the RNA transcript level using RT-PCR (*n* = 2 infection replicates). The expression of the inverted FlipArray was assessed at multiple timepoints following EFS-Cre infection using sequence-specific primers for the inverted FlipArray transcript as normalized to the Cpf1 mRNA level. **e**, **f** Representative Illumina targeted amplicon sequencing of the crNf1 target site (**e**) and crPten target site (**f**) in uninfected controls. **g**, **h** Representative Illumina targeted amplicon sequencing of the crNf1 target site (**g**) and crPten target site (**h**) 7 days after infection with lentivirus containing EFS-Cpf1-puro; U6-NPF-FlipArray. Where relevant, the top 5 most frequent variants are shown, with the associated variant frequencies in the boxes to the right. **i**, **j** Representative Illumina targeted amplicon sequencing of the crNf1 target site (**i**) and crPten target site (**j**) 17 days after infection with lentivirus containing EFS-Cpf1-puro; U6-NPF-FlipArray and 10 days following EFS-Cre infection. Where relevant, the top five most frequent variants are shown, with the associated variant frequencies in the boxes to the right. **k** Dot plot detailing the total variant frequencies at the crNf1 and crPten target sites in uninfected cells (red), 7 days after FlipArray transduction (−Cre) (green), and 17 days after FlipArray transduction (+Cre) (blue). *n* = 2 cell replicates for uninfected group, *n* = 3 for other conditions. All error bars are mean ± s.e.m

To detect Cre-mediated inversion of the FlipArray, we isolated genomic DNA from the NPF-expressing lung cancer cells before infection with EFS-Cre and 10 days after infection. We designed primers that would only generate a product if the FlipArray had successfully inverted (Fig. 2a). We also designed primers specific for the non-inverted FlipArray. These data demonstrated robust FlipArray inversion (Fig. 2b). Specifically, by D10 following EFS-Cre, the FlipArray inversion frequency was 79.07 ± 8.23% (mean ± s.e.m.) (Fig. 2c). In order to monitor the induction of functional FlipArray inversion at the transcript level, we also isolated total RNA from the double-infected KPD cells at various timepoints. After cDNA synthesis, we utilized inversion-specific primers to detect inverted crRNA FlipArray transcripts (Fig. 2d). The induction of inverted FlipArray transcripts steadily increased through the course of the experiment, illuminating the kinetics of Cre-mediated inversion of the FlipArray and its subsequent transcription. The low-levels of inverted FlipArray transcripts at baseline could be due to spontaneous inversion, or an artifact of the primer design.

We sequenced the target sites of crNf1 and crPten to determine whether the NPF construct had indeed created mutations in a controlled stepwise manner. Uninfected controls did not have any significant variants at crNf1 (Supplementary Data 1) or crPten target sites (Supplementary Data 2) (Fig. 2e, f, k). 7 days following the first lentiviral infection with EFS-Cpf1-Puro; U6-NPF, indels were found at the crNf1 target site, but not the crPten site (Fig. 2g, h, k). Since the second crRNA is not transcribed prior to Cre recombination, this result affirms that inversion of NPF has not yet occurred at this time point. After another 10 days following infection with EFS-Cre lentivirus (17 days following the initial infection with EFS-Cpf1-Puro; U6-NPF), indels were found at both crNf1 and crPten target sites at high frequencies (Supplementary Data 3; Fig. 2i–k).

**Fig. 3:**
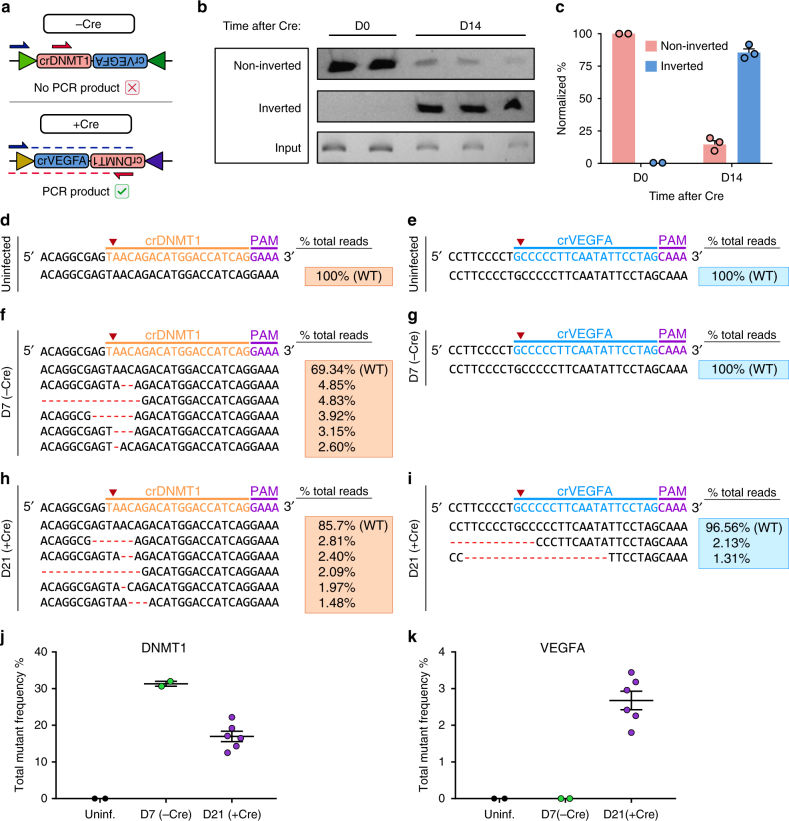
Inducible sequential mutagenesis in human cells through Cpf1-Flip. **a** Schematic of a FlipArray targeting human *DNMT1* and *VEGFA*. In the absence of Cre, crDNMT1 is expressed. Cre administration leads to the inversion of the FlipArray, leading to the expression of crVEGFA. **b** PCR-based detection of non-inverted and inverted FlipArrays at D0 (*n* = 2) and D14 (*n* = 3) following Cre, along with the input control. **c** Quantification of gel intensities in **b**, normalized to input and expressed as a percentage of total FlipArray abundance. **d**, **e** Representative Illumina targeted amplicon sequencing of the crDNMT1 target site (**d**) and crVEGFA target site (**e**) in uninfected controls. **f**, **g** Representative Illumina targeted amplicon sequencing of the crDNMT1 target site (**f**) and crVEGFA target site (**g**) 7 days after infection with lentivirus containing EFS-Cpf1-puro; U6-DVF-FlipArray. Where relevant, the top five most frequent variants are shown, with the associated variant frequencies in the orange box to the right. **h**, **i** Representative Illumina targeted amplicon sequencing of the crDNMT1 target site (**h**) and crVEGFA target site (**i**) 21 days after infection with lentivirus containing EFS-Cpf1-puro; U6-DVF-FlipArray and 14 days following EFS-Cre infection. The top five most frequent variants are shown, with the associated variant frequencies in the orange box to the right. **j**, **k** Dot plot detailing the total variant frequencies at the crDNMT1 and crVEGFA target sites in uninfected cells, 7 days after FlipArray transduction (−Cre), and 21 days after FlipArray transduction (+Cre). *n* = 2 cell replicates for uninfected and D7 condition, *n* = 6 for D21 condition. All error bars are mean ± s.e.m

To further demonstrate the utility of Cpf1-Flip in diverse biological systems, we designed a FlipArray targeting two human genes, *DNMT1* and *VEGFA*. The crRNA in the first position targets *DNMT1* (crDNMT1) while the second, inverted crRNA targets *VEGFA* (crVEGFA) (crDNMT1-crVEGFA FlipArray, or DVF) (Fig. 3a). Cre activation induces recombination of the *lox66*/*lox71* sites, such that crVEGFA becomes expressed. We transduced human HEK293T cells with EFS-Cpf1; U6-DVF lentivirus, followed by puromycin selection. To assess the functionality of the FlipArray, we then infected the cells with EFS-Cre lentivirus. Using primers specific to the non-inverted or inverted DVF FlipArray, we confirmed that Cre administration drives efficient inversion (Fig. 3b). In this system, inversion efficiency was 85.42 ± 2.90% by 2 weeks following EFS-Cre (Fig. 3c).

Next, to determine whether the Cpf1-Flip system had enabled sequential mutagenesis at the crDNMT1 and crVEGFA target sites, we performed deep sequencing. As anticipated, uninfected controls did not have significant mutations at either site (Supplementary Data 4, 5; Fig. 3d, e, j, k). Seven days after transduction with EFS-Cpf1; U6-DVF lentivirus, significant indels were found at the crDNMT1 target site but not at the crVEGFA target locus (Fig. 3f, g, j, k). The cells were then infected with EFS-Cre to cause FlipArray inversion, leading to expression of crVEGFA. 21 days after the initial transduction (14 days after EFS-Cre administration), significant indels were observed at both crDNMT1 and crVEGFA target sites (Supplementary Data 6; Fig. 3h–i). In these data, the *DNMT1* cutting efficiency appeared to be consistently lower at D21 than at D7. This is likely a consequence of random sampling, as only a subset of the D7 cells were subsequently taken forward for Cre infection. In addition, it is possible that *DNMT1* loss affects cell viability, given its crucial role in maintaining DNA methylation^27^. The cutting efficiency at crVEGFA was notably lower compared to crDNMT1. This contrast may be due to lower efficiency of the crRNA itself, as well as inefficiencies in FlipArray expression or subsequent crRNA array processing. Taken together, these results demonstrate that Cpf1-Flip is a flexible tool for sequential mutagenesis based on the Cpf1:crRNA complex, temporally controlled by Cre recombinase.

### Modeling acquired resistance to immunotherapy with Cpf1-Flip

**Fig. 4:**
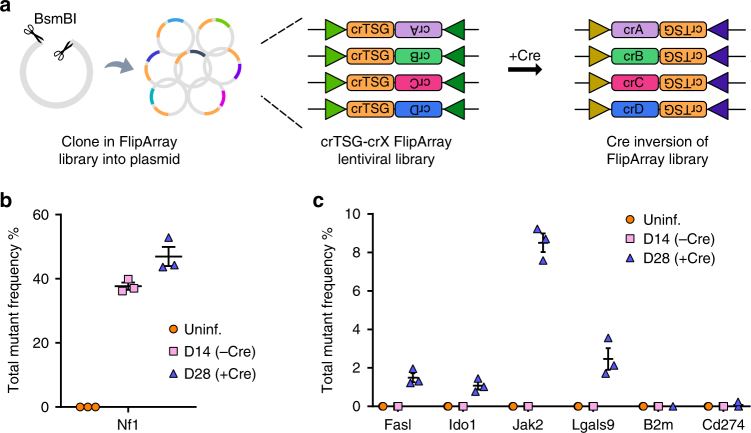
Pooled sequential mutagenesis to model acquired resistance to immunotherapy. **a** Schematic of the experimental approach for pooled sequential mutagenesis using Cpf1-Flip. Following restriction digest, a library of FlipArrays is cloned into the base vector. In each FlipArray, the first crRNA targets a tumor suppressor (*Nf1*), while the second crRNA targets a panel of putative immunomodulatory factors. Cre-mediated inversion induces expression of the second crRNA. **b** Dot plot detailing the total variant frequencies at the crNf1 target site in uninfected cells, 14 days after FlipArray transduction (−Cre), and 28 days after FlipArray transduction (+Cre). **c** Dot plot detailing the total variant frequencies at the second crRNA target sites (*Fasl*, *Ido1*, *Jak2*, *Lgals9*, *B2m*, and *Cd274*) in uninfected cells, 14 days after FlipArray transduction (−Cre), and 28 days after FlipArray transduction (+Cre). All error bars are mean ± s.e.m (*n* = 3 cell replicates for all conditions).

We next sought to apply Cpf1-Flip to model acquired resistance to immunotherapy in breast cancer cells (E0771 cell line). We designed a small pool of FlipArrays in which the first crRNA targets *Nf1* while the inverted second crRNA targets a panel of immunomodulatory factors (*Cd274*, *Ido1*, *B2m*, *Fasl*, *Jak2*, and *Lgals9*; referred to as TSG-Immune FlipArray library). These factors are thought to influence anti-tumor immunity and have been implicated in acquired resistance to checkpoint inhibitors. After pooled lentiviral transduction of E0771 cells with the TSG-Immune FlipArray library, we infected the cells with EFS-Cre lentivirus to induce FlipArray inversion (Fig. 4a). Upon Cre-mediated inversion, the second crRNA is expressed and triggers the knockout of various immunomodulatory factors, thus mimicking the sequential evolution of cancers in the face of immunotherapeutic pressures.

Targeted amplicon sequencing confirmed efficient mutagenesis of *Nf1* (Fig. 4b), followed by mutagenesis of the immunomodulatory factors upon Cre-mediated FlipArray inversion (Fig. 4c) (Supplementary Data 7, 8). Given the pooled nature of these experiments, lower population-level cutting efficiencies are anticipated at the second loci, as only a sixth of the total cell population, on average, is infected with a given FlipArray. The lack of consistent mutagenesis at the crB2m and crCd274 target sites may be intrinsic to the crRNA sequences themselves, a result of inefficient Cre infection/recombination and FlipArray processing, or simply a consequence of biased representation within the cell pool. Of note, we observed high cutting efficiencies at the *Jak2* locus despite the pooled nature of the experiment. Since these cells were processed completely in parallel as a minipool, the observation that crJak2 and crLgals9 showed consistent mutagenesis points to intrinsic differences in crRNA targeting efficiencies as the key factor underlying the lack of consistent cutting by crB2m and crCd274. Collectively, these data demonstrate the potential of Cpf1-Flip to facilitate sequential genetic screens—for instance, to model the acquisition of resistance mutations to cancer immunotherapy.

## Discussion

We introduced Cpf1-Flip, an inducible sequential mutagenesis system using invertible crRNA FlipArrays. As a proof-of-concept, we demonstrated sequential mutagenesis in both mouse and human cells, while additionally performing pooled sequential mutagenesis in a cancer cell line. These data revealed that the cutting efficiency of the second target loci can be low with certain crRNAs despite successful FlipArray inversion. The most likely explanation for the discordance between FlipArray inversion and subsequent mutagenesis of the second target locus is the differing efficiencies of the crRNAs themselves. This is corroborated by the variance observed across independent crRNAs in the pooled TSG-Immune library (Fig. 4), where consistent cutting efficiencies were observed at the *Jak2* and *Lgals9* target sites, but not at *B2m* or *Cd274* sites. Moreover, cells with different crRNAs in a pool can undergo random drift or selection, further diverting their relative fractions and thereby indel frequencies. Nevertheless, the FlipArray library can be readout by barcoded PCR of the specific crRNA cassette followed by high-throughput sequencing. Thus, as with all CRISPR screens, pooled screen studies using Cpf1-Flip would require multiple independent FlipArrays targeting each gene/gene pair to ensure fair representation in the mutant pool. In the future, the use of optimized crRNA sequences^28^, improved FlipArray designs, and engineered Cpf1 enzymes will improve the consistency and efficiency of Cpf1-Flip.

By altering the composition and length of the crRNA arrays within the FlipArray, one could readily engineer more complex CRISPR perturbation programs. Designs with two or more crRNAs within an invertible FlipArray at baseline would empower stepwise double knockouts (2 + 2, or quadruple knockouts as an end result) or higher dimensional sequential mutagenesis. Of note, the use of modified Cre systems such as CreER^29^, photoactivatable Cre^30^, and split-Cre^31^ would provide even greater control of FlipArray inversion. Utilizing orthogonal recombinases and recognition sites in the crRNA array would allow for even more complex multi-step gene editing programs. Through the use of tethered Cpf1 variants, FlipArrays could also be potentially used for sequential and reversible gene activation^32–34^, repression^35–37^, or epigenetic modification^38–40^ (Supplementary Fig. 1a). Given the scalability and flexibility of FlipArrays, conditional genetic studies for phenotypes that only emerge upon sequential genetic events can be performed using Cpf1-Flip either in culture or in vivo (Supplementary Fig. 1b). Since new mutations are stochastically acquired by rare individual cells within tumors^41^, Cpf1-Flip could therefore be relevant for studying the dynamics of rare tumor subclones under varying selection pressures, such as immunotherapy.

All such applications of Cpf1-Flip and its derivatives could potentially be self-contained within a single viral vector, facilitating direct in vivo sequential genetic manipulations and functional studies.

## Methods

### FlipArray design and construction

The empty EFS-Cpf1-Puro; U6-FlipArray vector was constructed by modification of the pY109 lentiviral vector (Addgene plasmid #84740)^16^. After BsmbI digestion (FastDigest Esp3I, ThermoScientific) to linearize the U6 crRNA expression cassette, oligo cloning was performed to insert a *lox66* sequence, a DR, two BsmbI sites, and an inverted *lox71*. The empty vector thus expresses LbCpf1 and puromycin resistance from an EFS promoter, while a U6 promoter drives expression of a *lox66*/*lox71* flanked crRNA expression module containing two BsmbI sites. BsmbI digestion and oligo cloning was then used to insert FlipArrays into the empty vector. For a given pair of crRNAs, the following oligo overhangs were used for cloning:

Oligo1 5′ overhang: TAGAT

Oligo1 3′ overhang: A

Oligo2 5′ overhang: GTTAT

Oligo2 3′ overhang: A

The main body of the FlipArray was structured as such:

5′-crRNA 1–6xT–6xA–Rev.Complement(crRNA 2)–Rev.Complement(DR)-3′

In this study, the following oligo sequences were used to target *Nf1* and *Pten*:

crNf1 spacer: TAAGCATAATGATGATGCCA

crPten spacer: TGCATACGCTATAGCTGCTT

NPF oligo 1 (to clone into vector): TAGATTAAGCATAATGATGATGCCATTTTTTAAAAAAAAGCAGCTATAGCGTATGCAATCTACACTTAGTAGAAATTAA

NPF oligo 2 (to clone into vector): GTTATTAATTTCTACTAAGTGTAGATTGCATACGCTATAGCTGCTTTTTTTTAAAAAATGGCATCATCATTATGCTTAA

The following crRNA spacer sequences were also used, with analogous oligo designs for cloning into the Cpf1-Flip vector:

crDNMT1: CTGATGGTCCATGTCTGTTA

crVEGFA: CTAGGAATATTGAAGGGGGC

crFasl: GTCCGGCCCTCTAGGCCCAC

crIdo1: CTACAGGGAATGCACAGATG

crJak2: ACATACATCGAGAAGAGTAA

crLgals9: TGCAGTACCAACACCGCGTA

crB2m: TGCACGCAGAAAGAAATAGC

crCd274: TAAAGCACGTACTCACCGAG

### Lenti-Cre vector design and construction

The Lenti-Cre vector was designed to express the Cre recombinase under a constitutive EFS promoter. The plasmid was generated by PCR amplification of Cre and EFS fragments followed by Gibson assembly into a previous lentiviral vector backbone (lentiGuidePuro)^42^.

### Cell culture and genomic DNA extraction

KPD cells^26,43^, E0771 cells (CH3 BioSystems), and HEK293T cells (ThermoFisher) were cultured in DMEM supplemented with 10% FBS and 1% penicillin/streptomycin. Experiments were conducted with at least two independent cellular replicates. For genomic DNA extraction, ~500,000 cells were isolated. Cells were spun down at 500 × *g* for 5 min and washed once with 1×PBS. After removing the supernatant, cell pellets were resuspended in 500 µl QuickExtract DNA Extraction Solution (Epicentre). Cells were then incubated at 65 °C for 20 min, followed by incubation at 85 °C for 5 min to deactivate the enzymes.

### Detection of FlipArray inversion by genomic DNA PCR

The following primers were used to amplify the U6 cassette from genomic DNA:

RdF: GAGGGCCTATTTCCCATGATTCCTTCATATTT

RdR: ACAGTGCAGGGGAAAGAATAGTAGA

PCR conditions: 98 °C 2 min, 32 cycles of (98 °C 1 s, 62 °C 5 s, 72 °C 15 s), 72 °C 2 min, 4 °C hold.

Following Qiagen PCR purification, 2 ng of the first PCR were used for the second inversion-specific or non-inverted-specific PCR. The following primers were used for detection of non-inverted or inverted FlipArrays:

NPF_F: TCTTGTGGAAAGGACGAAACACCG

NPF_R: TGCATACGCTATAGCTGCTTTTTTTTAAAAAATGGCA

NPF_R_inv: TAAGCATAATGATGATGCCATTTTTTAAAAAAAAGCAG

DVF_F: TCTTGTGGAAAGGACGAAACACCG

DVF_R: GGGCTTTTTTAAAAAATAACAGACATGGACCATCAG

DVF_R_inv: CTGATGGTCCATGTCTGTTATTTTTTAAAAAAGCCC

PCR conditions: 98 °C 2 min, 14 cycles of (98 °C 1 s, 62°C 5 s, 72 °C 2 s), 72 °C 2 min, 4 °C hold. PCR reactions specific to non-inverted and inverted FlipArrays were performed and analyzed simultaneously for each sample. Quantification was done on 2% E-gel using low-range quantitative ladder (ThermoFisher), and was normalized to the first PCR product. Full gel images are shown in Supplementary Fig. 2a–c.

### Quantification of inverted FlipArray transcripts

KPD cells were cultured in DMEM supplemented with 10% FBS and 1% penicillin/streptomycin. For RNA extraction, ~200,000 cells were isolated and spun down at 500 × *g* for 5 min. After a PBS wash, cells were resuspended in 450 µl TRIzol. 100 µl of chloroform was then added to each tube, followed by rigorous vortexing for 15 s and centrifuging at 12,000 × *g* for 10 min. The supernatant containing RNA was then purified using a Qiagen RNeasy Kit following the RNA cleanup protocol. cDNA was generated by reverse transcription with random hexamers. PCR detection of inverted crRNA FlipArray transcripts was done using the following primers:

Inv_FlipArray_F: TGTAGATAGCGCTATAACTTCGTATAGC

Inv_FlipArray_R: AAGCAGCTATAGCGTATGCAATC

PCR conditions: 98 °C 2 min, 34 cycles of (98 °C 1 s, 56°C 5 s, 72°C 5 s), 72 °C 2 min, 4 °C hold.

As a normalization control, PCR detection of Cpf1 transcripts was done using the following primers:

Cpf1_F: TTCTTTGGCGAGGGCAAGGAGACAA

Cpf1_R: GCACGCGCACCTCTGTATTGATCTT

PCR conditions: 98 °C 2 min, 40 cycles of (98 °C 1 s, 56 °C 5 s, 72 °C 20 s), 72 °C 2 min, 4 °C hold.

Quantification of inverted FlipArray RNA abundance was done on 2% E-gel using low-range quantitative ladder (ThermoFisher), and was normalized to Cpf1 mRNA transcript abundance.

### Detection of Cpf1 mutagenesis

The genomic regions flanking the crRNA target sites were amplified from genomic DNA using the following primers:

Nf1_F: GGGTCCGATTGCCAGTACCC

Nf1_R: AACGTGCACCTCCCTTGTCA

Pten_F: ACTCACCAGTGTTTAACATGCAGGC

Pten_R: GGCAAGGTAGGTACGCATTTGCT

DNMT1_F: CTGGGACTCAGGCGGGTCAC

DNMT1_R: CCTCACACAACAGCTTCATGTCAGC

VEGFA_F: CTCAGCTCCACAAACTTGGTGCC

VEGFA_R: AGCCCGCCGCAATGAAGG

Cd274_F: GAATGGTCCCCAAGACAAAGAAGAAGA

Cd274_R: ATTCCCAAAGGAGAACCTGTAATGAGC

Ido1_F: TTCATTGTTCTTCACCCCATGATTGGT

Ido1_R: CCCATGACTTTCCTAAGGAGTGTGAAA

B2m_F: TGTCAGGTGGAGTCTAGTGGTAGAAAA

B2m_R: ATTGGGCACAGTGACAGACTTCAATTA

Fasl_F: CGCCTGATTCTCCAACTCTAAAGAGAC

Fasl_R: GCAAAGAGAAGAGAACAGGAGAAAGGT

Jak2_F: AGATTCATAGCTGTCGTTCATCACTGG

Jak2_R: GTTAGTTCTCTTTCTGCTTCTCTGCCA

Lgals9_F: TTTGGCATCTTCACCAAGGTAGATTGT

Lgals9_R: TAAGCCTGGACTAAGTAAGTGAATGCC

PCR conditions: 98 °C 2 min, 32 cycles of (98 °C 1 s, 63 °C 5 s, 72 °C 20 s), 72 °C 2 min, 4 °C hold.

The genomic DNA from ~1000 cells was used for PCR with the NPF and DVF FlipArrays. For the TSG-Immune FlipArray library experiments, genomic DNA from ~6000 cells were used to account for the pooled nature of the experiment. The resultant PCR products were used for Nextera library preparation following manufacturer protocols. Reads were mapped to the mm10 or hg38 genome using BWA-MEM^44^, with settings -t 8 -w 200. After identification of indel variants using the pileup2indel function in VarScan v2.3.9, a 1% variant frequency threshold was to identify high confidence variants for NPF and DVF experiments. A less stringent 0.2% variant frequency threshold was used for the TSG-Immune experiments due to their pooled nature. All variant calls are detailed in Supplementary Data.

### Sample size determination

No specific methods were used to predetermine sample size.

### Blinding statement

Investigators were blinded for sequencing data analysis with generic sample IDs, but not blinded for PCR or RT-PCR.

### Code availability

Custom scripts will be available to the academic community upon reasonable request

### Data availability

The FlipArray base vector has been submitted to Addgene (ID: 109349) and is available to the academic community. Cell lines, and additional data will be available to the academic community upon reasonable request. The genomic sequencing data sets generated during the current study are available in NCBI SRA under accession SRP136201.

## Electronic supplementary material

Supplementary Information

Description of Additional Supplementary Files

Supplementary Data 1

Supplementary Data 2

Supplementary Data 3

Supplementary Data 4

Supplementary Data 5

Supplementary Data 6

Supplementary Data 7

Supplementary Data 8
