# Supplementary material for: Programmable sequential mutagenesis by inducible Cpf1 crRNA array inversion
Source: Nat Commun. 2018 May 15;9:1903. doi: 10.1038/s41467-018-04158-z (PMC5954137; doi:10.1038/s41467-018-04158-z)
Supplement: Supplementary file 1 — Supplementary Information [file 41467_2018_4158_MOESM1_ESM.pdf]

Supplementary Information for

Programmable sequential mutagenesis by inducible Cpf1 crRNA array inversion

Chow et al.

Supplementary Figure 1: Applications and variations of Cpf1-Flip

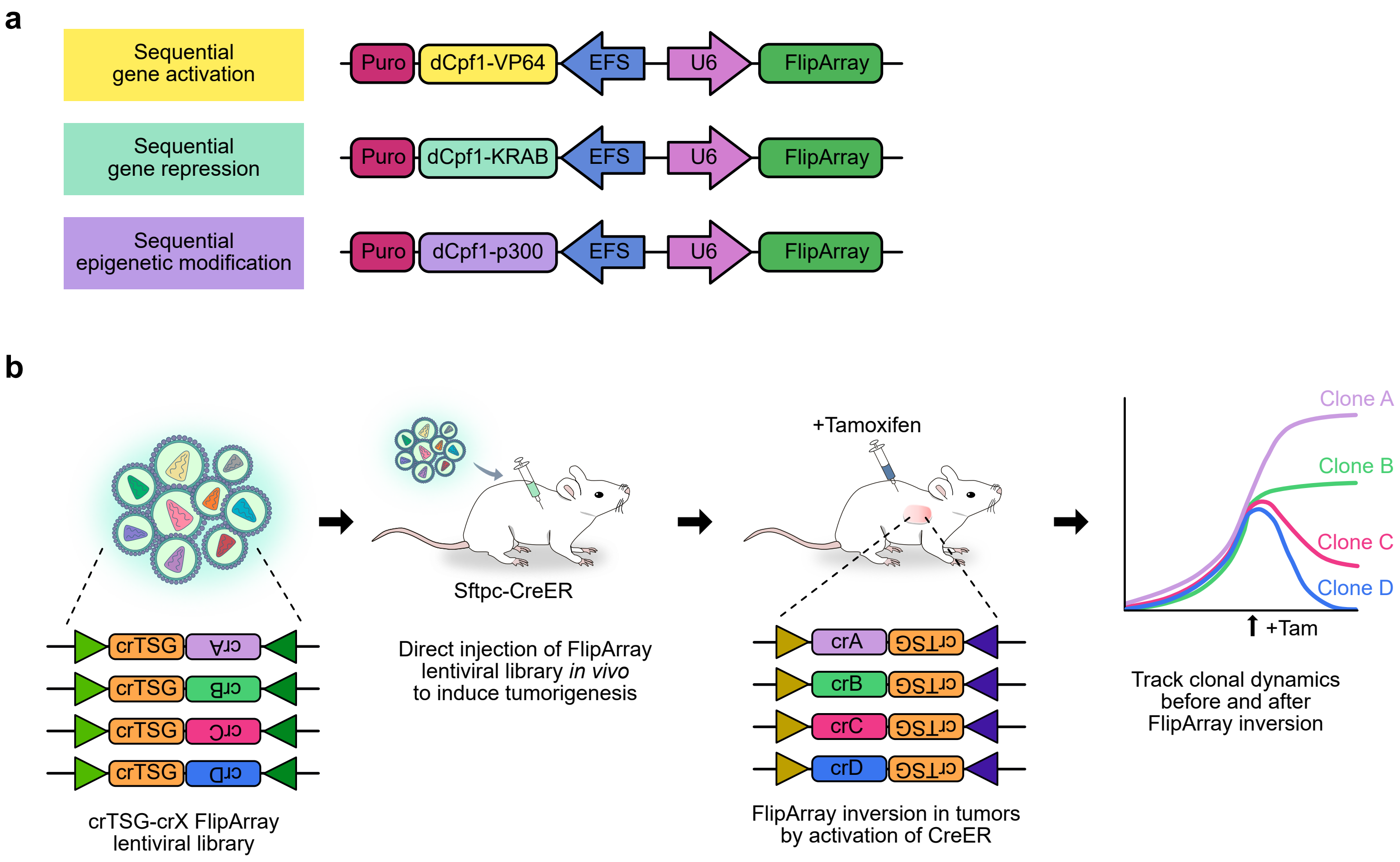

a. Schematic of several variations of Cpf1-Flip, using modified Cpf1 effector proteins. Sequential gene activation, gene repression, and epigenetic modification can all be readily performed using Cpf1-Flip.

b. Cpf1-Flip applied to model the evolution of cancer in a direct in vivo system. Since Cpf1-Flip operates in a stepwise manner, it is possible to temporally separate the initial mutagenesis event (in this case targeting a tumor suppressor gene, or TSG). After tumorigenesis, induction of FlipArray inversion activates the second set of crRNAs, allowing for parallel interrogation of clonal dynamics in vivo.

Supplementary Figure 2: Gel images

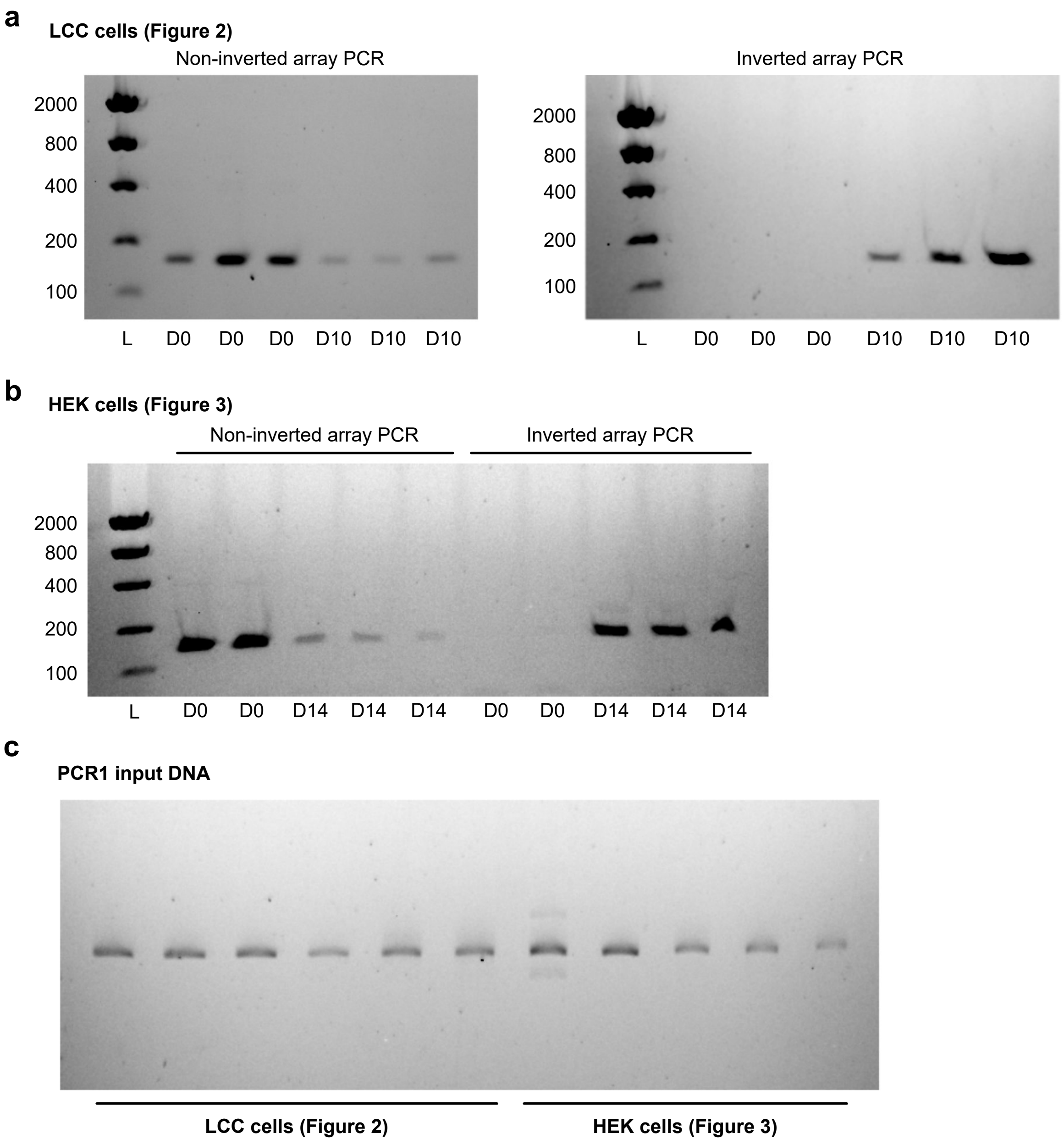

a. Gel image from Figure 2 for non-inverted array PCR (left) and inverted array PCR (right).

b. Gel image from Figure 3 for non-inverted array PCR (left) and inverted array PCR (right).

c. Input DNA from PCR1 used for non-inverted or inverted array PCR in **(a-b)**.
