# Supplementary material for: Programmable sequential mutagenesis by inducible Cpf1 crRNA array inversion
Source: Nat Commun. 2018 May 15;9:1903. doi: 10.1038/s41467-018-04158-z (PMC5954137; doi:10.1038/s41467-018-04158-z)
Supplement: Supplementary file 2 — Description of Additional Supplementary Files [file 41467_2018_4158_MOESM2_ESM.pdf]

Description of Additional Supplementary Files

File Name: Supplementary Data 1
Description: All unfiltered variant calls of Illumina targeted amplicon sequencing of the crNf1 target site in uninfected controls, 7 days after infection with lentivirus containing EFS-Cpf1-puro; U6-NPFFlipArray, and 17 days after infection with lentivirus containing EFS-Cpf1-puro; U6-NPF-FlipArray (10 days following EFS-Cre infection). (related to Fig. 2)

File Name: Supplementary Data 2
Description: All unfiltered variant calls of Illumina targeted amplicon sequencing of the crPten target site in uninfected controls, 7 days after infection with lentivirus containing EFS-Cpf1-puro; U6-NPFFlipArray, and 17 days after infection with lentivirus containing EFS-Cpf1-puro; U6-NPF-FlipArray (10 days following EFS-Cre infection). (related to Fig. 2)

File Name: Supplementary Data 3
Description: Total mutant frequencies at the crNf1 and crPten target sites in uninfected controls, 7 days after infection with lentivirus containing EFS-Cpf1-puro; U6-NPF-FlipArray, and 17 days after infection with lentivirus containing EFS-Cpf1-puro; U6-NPF-FlipArray (10 days following EFS-Cre infection). (related to Fig. 2)

File Name: Supplementary Data 4
Description: All unfiltered variant calls of Illumina targeted amplicon sequencing of the crDNMT1 target site in uninfected controls, 7 days after infection with lentivirus containing EFS-Cpf1-puro; U6-DVFFlipArray, and 21 days after infection with lentivirus containing EFS-Cpf1-puro; U6-DVF-FlipArray (14 days following EFS-Cre infection). (related to Fig. 3)

File Name: Supplementary Data 5
Description: All unfiltered variant calls of Illumina targeted amplicon sequencing of the crVEGFA target site in uninfected controls, 7 days after infection with lentivirus containing EFS-Cpf1-puro; U6-DVFFlipArray, and 21 days after infection with lentivirus containing EFS-Cpf1-puro; U6-DVF-FlipArray (14 days following EFS-Cre infection). (related to Fig. 3)

File Name: Supplementary Data 6
Description: Total mutant frequencies at the crDNMT1 and crVEGFA target sites in uninfected controls, 7 days after infection with lentivirus containing EFS-Cpf1-puro; U6-DVF-FlipArray, and 21 days after infection with lentivirus containing EFS-Cpf1-puro; U6-DVF-FlipArray (14 days following EFS-Cre infection). (related to Fig. 3)

File Name: Supplementary Data 7
Description: All unfiltered variant calls of Illumina targeted amplicon sequencing of the TSGImmune target sites in uninfected controls, 14 days after infection with lentivirus containing EFS-Cpf1-puro; U6-TSG-Immune-FlipArray library, and 28 days after infection with lentivirus containing EFS-Cpf1-puro; U6- TSG-Immune-FlipArray library (14 days following EFS-Cre infection). (related to Fig. 4)

File Name: Supplementary Data 8
Description: Total mutant frequencies at the crNf1 and TSG-Immune target sites in uninfected controls, 7 days after infection with lentivirus containing EFS-Cpf1-puro; U6-TSG-Immune FlipArray library, and 28 days after infection with lentivirus containing EFS-Cpf1-puro; U6- TSG-Immune FlipArray library (14 days following EFS-Cre infection). (related to Fig. 4)
